# The Protective Effect of Different Polar Solvent Extracts of Er Miao San on Rats with Adjuvant Arthritis

**DOI:** 10.1155/2020/5305278

**Published:** 2020-02-20

**Authors:** Wei Zhang, Qiying Zhang, Zihua Xuan, Juan Liang, Dongping Yang, Meihuizi Ding, Housheng Zhu, Bo Su, Xing Dai, Xiaoyi Jia

**Affiliations:** ^1^School of Pharmacy, Anhui University of Chinese Medicine, Hefei 230012, China; ^2^Anhui Province Key Laboratory of Traditional Chinese Medicine Decoction Pieces of New Manufacturing Technology, Hefei 230012, China; ^3^Anhui Province Key Laboratory of Chinese Medicinal Formula, Hefei 230012, China; ^4^The First Clinical Medical College, Anhui Medical University, Hefei 230032, China

## Abstract

**Objective:**

The aim of this study was to evaluate the antiarthritic effects of different polar solvent extracts of Er Miao San (EMS) on model rats with adjuvant arthritis (AA) and screen the effective pats of EMS in the treatment of arthritis.

**Methods:**

Four different polar solvent extracts of EMS such as petroleum ether (PE), methylene chloride (CH_2_Cl_2_), ethyl acetate (EtOAc), and *n*-butanol (*n*-butanol (

**Results:**

Administration of EtOAc and CH_2_Cl_2_ parts remarkably inhibited the paw swelling, decreased the index of arthritis, decreased the body weight loss, and improved the changes of histopathology. Furthermore, the concentrations of proinflammatory cytokines (TNF-*α*, IL-1*β*, and IL-6) were significantly lower, while the anti-inflammatory cytokine (IL-10) was remarkably higher compared with that in the model group. And the result of UHPLC analysis indicated that the effective parts of EMS contain berberine and atractylodin.

**Conclusions:**

EtOAc and CH_2_Cl_2_ are the effective parts of EMS that can improve arthritis. In particular, berberine and atractylodin may be responsible for the antiarthritic activity of EMS. This research provided pharmacological and chemical foundation for the application of EMS in treating rheumatoid arthritis (RA).

## 1. Introduction

Er Miao San (EMS), formerly known as changzu powder, comes from Zhenheng Zhu (dan xi xin fa), which is composed of equal amounts of Phellodendri Cortex (PC) and Atractylodis Rhizoma (AR). PC contains various chemical derivatives, such as berberine and jatrorrhizine, which were proven to have anti-inflammatory effects, antimicrobial effects, antitumor effects, antiulcer effects, antioxidant effects, and so on [1]. AR contains atractylodin, *β*-eudesmol, hinesol, and atractylone, which have anti-inflammatory activities, anticancer activities, pharmacological activities on the nervous system, gastrointestinal system, and cardiovascular system, and so on [2]. PC is bitter in taste and cold in nature. AR is pungent and bitter in taste and hot in nature. The effect of clearing heat, removing dryness, and dampness is more significant after combining the two herbs. Previous research in our group showed that EMS has a good anti-inflammatory effect on the acute inflammatory model induced by carrageenan in rats. Furthermore, EMS attenuated complete Freund's adjuvant-induced arthritis (AA) via regulation of Th17/Treg cells. However, the effective components of EMS against rheumatoid arthritis (RA) are not clear, and few studies have been conducted. RA is a chronic systemic autoimmune disease characterized by synovitis of the joints [3–5]. The rat model of AA has pathological changes similar to human RA [6]. The modeling method is simple and easy to use with good repeatability, which is widely used in the screening and mechanism research of antiarthritic drugs.

In this study, we aimed to investigate the effective parts of EMS for antiarthritic effects and revealed the possible molecular mechanism of action on the AA rat model, which will provide the foundation for further research on the anti-rheumatoid arthritic effect of EMS.

## 2. Materials and Methods

### 2.1. Animals

Male Sprague Dawley (SD) rats (weighting 180 ± 20 g) were purchased from the Experimental Animal Center of Anhui Medical University (Hefei, China). All rats were fed standard chow diet and water under a controlled temperature of 25°C and a 12 h light/12 h dark cycle in the animal room. All experiments were approved by the Ethics Review Committee for Animal Experimentation of Anhui University of Chinese Medicine (Hefei, China).

### 2.2. Materials

PC and AR were obtained from Anhui Puren Herbal Pieces Co., Ltd (Bozhou, Anhui Province, China). They were authenticated by Dr Liu SJ (School of Pharmacy, Anhui University of Chinese Medicine). Methotrexate (MTX) tablets were obtained from Xinyi Medical Limited Company (Shanghai, China). Liquid paraffin, petroleum ether, and *n*-butanol were purchased from Shanghai SuYi Chemical Reagent Co., Ltd. Methylene chloride was purchased from Shanghai Richjoint Chemical Reagents Co., Ltd. Ethyl acetate was purchased from Jiangsu Qiangsheng Function Chemistry Co., Ltd. ELISA kits for TNF-*α*, IL-10, IL-6, and IL-1*β* were purchased from 4A Biotech Co., Ltd. The reference substances of berberine (Batch no. 633-65-8) and atractylodin (Batch no. 55290-63-6) were purchased from National Institute for Identification of Pharmaceutical and Biological Products (Beijing, China) and Yuanbaofeng Medical Technology Co., Ltd. (Nanjing, China), respectively. The purities of the two standard compounds were more than 98%. All other reagents used were of analytical grade.

### 2.3. Preparation of EMS

For preparing EMS, equal parts of PC and AR (total 600 g) were mixed and crushed. EMS was decocted with boiling water three times for 1.5 h, 1.0 h, and 0.5 h, and then the suspension after the third time was collected and condensed by evaporation in a water bath. The obtained suspension was extracted by different polar solvents, and then four different polar extracts from the aqueous extract of EMS (PE part, CH_2_Cl_2_ part, EtOAc part, and *n*-BuOH part) were obtained.

### 2.4. Induction of AA Animal Model and Treatment

The AA model was constructed in SD rats as previously described [7]. In brief, complete Freund's adjuvant (CFA, 10 mg/mL) was emulsified with bacillus Calmette–Guerin (BCG) and dissolved in liquid paraffin. AA was induced by a single intradermal injection of 100 *μ*L CFA into the left hind metatarsal footpad of a rat. The normal group was injected the same amount of physiological saline. Rats were randomly assigned to seven groups, namely, normal control, model group, administered groups (PE part group, CH_2_Cl_2_ part group, EtOAc part group, and *n*-BuOH part group) (3 g/kg, calculated by the crude drug), and positive control group: MTX group (0.5 mg/kg). Administered (once per day, a total of 14 days) and MTX (every 3 days, a total of five times) groups were administered via gavage after immunization for 15 days. The normal and model groups were administered with an equal volume of carboxymethyl cellulose at the same time.

### 2.5. Evaluation of Arthritis

The right hind paw volume was measured with a volume meter (PV-200, Chengdu Technology Market Co., Ltd.) before immunization (basic value, 0 days) and after immunization on days 14, 18, 22, 26, and 30. The body weights of rats were recorded every week by an electronic scale. The polyarthritis index was scored according to the following criteria for each toe, with a maximum score of 16 points: 0, no swelling normally; 1, erythema and slight swelling of the ankle joint; 2, erythema and slight swelling of the ankle joint to the metatarsophalangeal or capsular joint; 3, erythema and moderate swelling of the ankle to the metatarsophalangeal or capsular joint; and 4, erythema and severe swelling of the ankle to the metatarsophalangeal joint.

### 2.6. Analysis of Cytokines (TNF-*α*, IL-6, IL-1*β*, and IL-10) in the Serum

After 24 hours of the last administration, the blood from the femoral artery was taken via anesthesia. The serum was collected by centrifugation at 2500 rpm for 8 minutes after standing for half an hour and frozen at −80°C until use. The levels of TNF-*α*, IL-1*β*, IL-6, and IL-10 were determined by the ELISA kit (4A Biotech Co., Ltd.), according to the manufacturer's instructions. The absorbance of each well was read at 450 nm with an ELISA plate reader (Thermo Scientific Multiskan Spectrum). Background absorbance of blank wells was subtracted from the standards and unknowns prior to determination of sample concentrations.

### 2.7. Histological Evaluation of the Ankle Joint

Rats were sacrificed via anesthesia after 24 hours of the last administration. The right (secondary) ankle joints were obtained and fixed with 4% paraformaldehyde. Then, they were decalcified in 10% ethylenediaminetetraacetic acid and embedded in paraffin for histopathological analysis. Serial paraffin sections were stained with hematoxylin and eosin (H&E). Pathological changes were evaluated under the double-blind condition with a light microscope. The severity of arthritis in the ankle joints was graded from 0 to 4 according to mononuclear cell infiltration, synovial proliferation, pannus formation, and cartilage and bone erosion as described previously [8]_._

### 2.8. UHPLC Analysis of EMS

The effective parts of EMS and reference substances of berberine and atractylodin were dissolved in methanol at appropriate concentration, respectively. A Waters ACQUITY H-CLASS system (America) was used for UHPLC. The sample was examined by a Discovery C18 analytical column (2.1 mm × 100 mm, 1.7 *μ*m particle size; Supelco, USA). Mobile phase A was acetonitrile, and mobile phase B consisted of 0.1% formic acid in water, and the gradient program was used as follows: 0–2 min, 8% A; 2–5 min, 8–12% A; 5–15 min, 12% A; and 15–25 min, 30–40% A. Chromatography analysis was performed at a flow rate of 0.2 mL/min and at a room temperature of 30°C. The detection wavelength was set at 284 nm, and the injection volume was 2 *μ*L. Under the condition of this chromatography, both the experimental sample and the control sample were analyzed and the results were obtained.

### 2.9. Statistical Analysis

Data obtained from the pharmacological experiment are expressed as mean ± SEM. Differences between the control and the treatments in this experiment were tested by one-way analysis of variance (ANOVA) using SPSS software. A probability of *P* < 0.05 was considered significant, and a probability of *P* < 0.01 was considered very significant.

## 3. Results

### 3.1. Effects of Different Polar Solvent Extracts of EMS on the Clinical Signs in AA Rats

**Figure 1:**
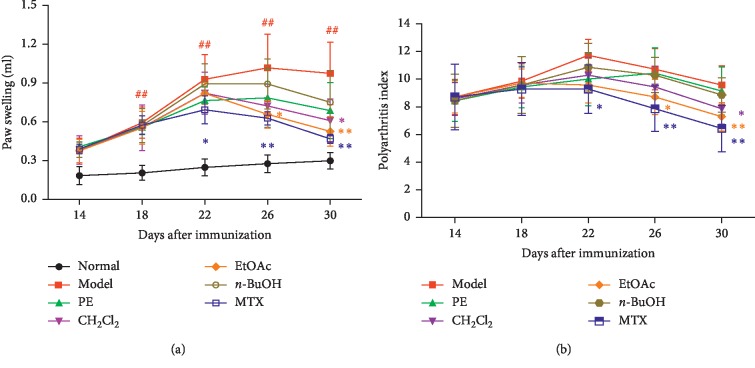
Treatment with different polar solvent extracts attenuates clinical signs in rats with AA. Effects of different polar solvent extracts of EMS on (a) paw swelling and (b) polyarthritis index in AA rats. ^##^*P* < 0.01 vs. normal; ^*∗*^*P* < 0.05 and ^*∗∗*^*P* < 0.01 vs. model (*n* = 7).

The paw swelling, arthritis score, and body weight loss of rats were determined to evaluate the severity of arthritis and the anti-inflammatory effect of the drug. After the inflammation, the rats ate less and moved more slowly. The secondary inflammatory reaction appeared around days 13–16 and peaked around days 20–23 after immunization. The body weight of the AA model slowly increased and was significantly less than that of the normal model (*P* < 0.05) (Table 1). Different polar solvent extracts of EMS treatment attenuated the severity of AA, as demonstrated by both the paw swelling and the polyarthritis index, in which EtOAc and CH_2_Cl_2_ parts were preferred (Figure 1). Also, MTX significantly attenuated clinical sings in rats with AA. The body weight of rats that underwent treatment with different polar solvent extracts of EMS was higher than that of the model group. However, no statistical difference was observed (*P* > 0.05) (Table 1).

### 3.2. Effects of Different Polar Solvent Extracts of EMS on Ankle Joint Histopathological Changes in AA Rats

**Figure 2:**
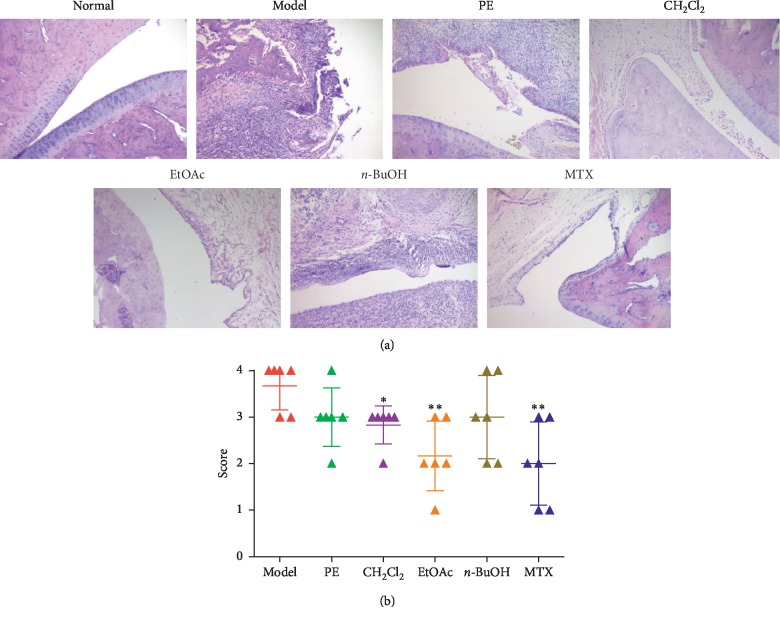
Effects of different polar solvent extracts of EMS on histopathological changes in AA rats. (a) Photographs of representative effects on the joint histopathology of AA rats (H&E, ×100). (b) Scores of histopathology in the rats. ^*∗*^*P* < 0.05 and ^*∗∗*^*P* < 0.01 vs. model (*n* = 5).

The histopathological index is the gold index of diagnosis. To further determine the antiarthritic effective part of EMS, the therapeutic effects of different polar solvent extracts of EMS on AA rats were evaluated by histological analysis. The results showed that AA rats developed severe arthritis, which was characterized with massive inflammatory cell infiltration of the synovial tissue, synovial proliferation, pannus formation, and articular cartilage and bone erosion and destruction. In accordance with the clinically observed effects on the severity of arthritis, treatment with different polar solvent extracts of EMS inhibited the histological damage, in which EtOAc and CH_2_Cl_2_ parts were preferred. MTX (0.5 mg/kg) also effectively inhibited these histological severity scores (Figure 2).

### 3.3. Effects of Different Polar Solvent Extracts of EMS on the Production of Serum Cytokines in AA Rats

**Figure 3:**
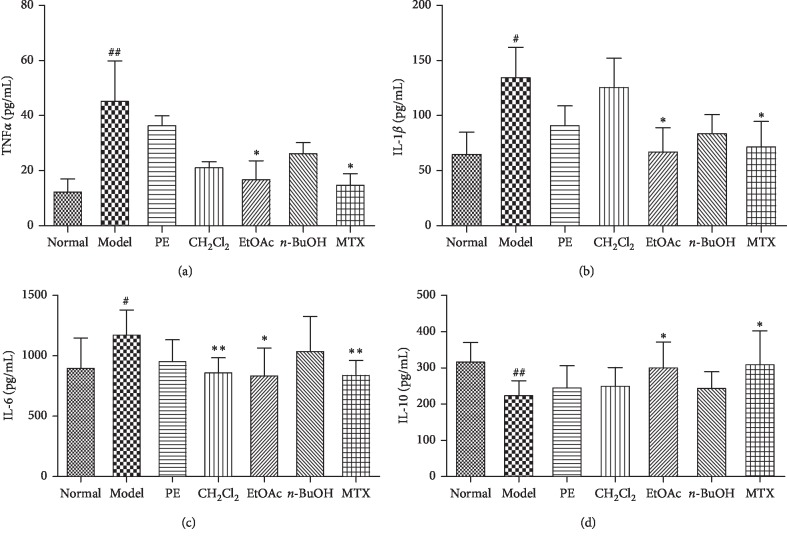
Effects of different polar solvent extracts of EMS on cytokine production in the serum of AA rats. The serum was collected, and the levels of TNF-*α* (a), IL-1*β* (b), IL-6 (c), and IL-10 (d) were measured by ELISA. ^#^*P* < 0.05 and ^##^*P* < 0.01 vs. normal; ^*∗*^*P* < 0.05 and ^*∗∗*^*P* < 0.01 vs. model (*n* = 4).

It is well known that the difference in proinflammatory and anti-inflammatory cytokine activity plays a vital role in RA pathogenesis. In order to clarify the mechanism of improvement of AA in different polar sites after treatment, the proinflammatory cytokines (TNF-*α*, IL-1*β*, and IL-6) and anti-inflammatory cytokine (IL-10) in the serum of AA rats were measured. Similar to previous studies, the levels of TNF-*α*, IL-1*β*, and IL-6 in the AA model group were significantly higher than those in the normal group. However, the level of IL-10 was significantly decreased in the AA model group. The parts of EtOAc and MTX groups significantly inhibited the production of proinflammatory cytokines (TNF-*α*, IL-1*β*, and IL-6) (Figures 3(a)–3(c)) and increased the production of the anti-inflammatory cytokine (IL-10) (Figure 3(d)), while the part of CH_2_Cl_2_ significantly decreased the level of IL-6 (Figure 3(c)). The PE part and *n*-BuOH part had no effect on these cytokines.

### 3.4. Phytochemical Analyses of Antiarthritic Effective Part of EMS by UHPLC

**Figure 4:**
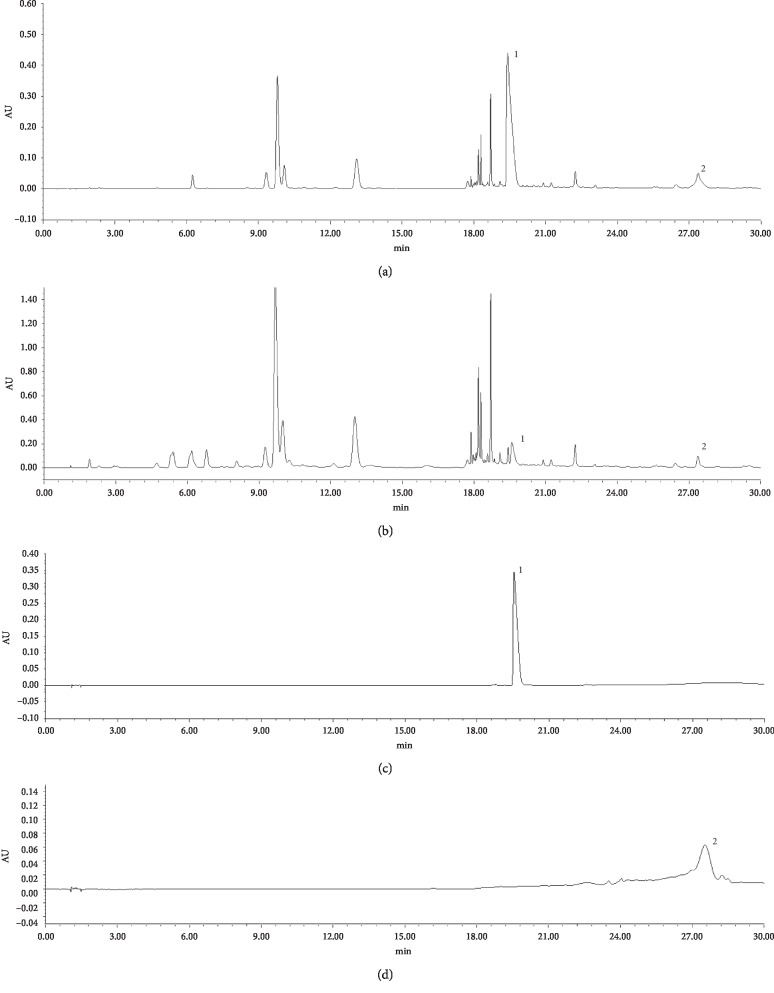
UHPLC analysis of the effective parts of EMS: (a) methylene chloride (CH_2_Cl_2_) part of EMS; (b) ethyl acetate (EtOAc) part of EMS; (c, d) reference substances: 1, berberine and 2, atractylodin.

According to Chinese Pharmacopoeia (Chinese Pharmacopoeia Commission, 2015), berberine and atractylodin are two compounds used to reflect the quality of PC and AR, respectively. Therefore, in this study, we also used these two compounds as control points to test whether the antiarthritic effective part of EMS contained them. As shown in Figure 4, the UHPLC chromatogram of reference compounds included berberine and atractylodin. The peaks of berberine and atractylodin of CH_2_Cl_2_ and EtOAc were identified by comparing the retention time with that of reference compounds. It means that the positions of CH_2_Cl_2_ and EtOAc clearly contain higher components of berberine and atractylodin, which can prove the anti-inflammatory effect of these two positions and provide an experimental reference for further research in the future.

## 4. Discussion

RA is one of the most common autoimmune diseases of joint damage [9–11]. It can severely impede the joint movement of patients and lead to physical disability, loss of working ability, decreased quality of life, and even premature death [12, 13]. Although the etiology and pathogenesis of RA are still unclear, inflammatory response plays an important role in the pathogenesis of RA. Therefore, anti-inflammatory therapy has become the main recommended strategy for treating RA. Traditional Chinese medicine has its unique advantages in the treatment of RA.

EMS as a compound for clearing away heat, detoxifying, and removing blood stasis has anti-inflammatory effect, improves blood circulation, and regulates the immune system. Clinical treatment of acute and chronic inflammatory diseases has shown significant efficacy. Previous researches in our group have shown that the aqueous extract of EMS can improve the symptoms of AA rats, but its effective part with anti-inflammatory property is not clear. In this study, we successively used different polar solvents to extract the aqueous extract of EMS. The four polar solvents are *n*-butanol, ethyl acetate, dichloromethane, and petroleum ether in order. According to the theory that similarities can be solvable easily in each other that the substances extracted from petroleum ether are lipid soluble compounds. Alkaloids, flavonoids, and other polar compounds may be extracted from ethyl acetate. Compounds with strong polarity, such as saponins and alkaloids, may be extracted from *n*-butanol, and dichloromethane is a lipophobic extraction solvent, which can extract most volatile oil and other substances. We investigated the antiarthritic effect of different solvent extracts of EMS on AA rats. The results showed that the EtOAc extract and CH_2_Cl_2_ extract of EMS were effective in inhibiting the swelling, reducing the arthritis score, and improving the histopathological changes of AA rats.

Cytokine TNF-*α* induces cascades of other inflammatory cytokines such as IL-1*β* and IL-6. It is an important proinflammatory factor in the inflammatory response and plays a key role in RA progression [14]. IL-1*β* is a small proinflammatory factor that belongs to the IL-1 family and has a wide range of biological effects. IL-1*β* receptor 1 (IL-1*β*-R1) can be expressed in different target cells. IL-1*β* can also induce the expression of other proinflammatory cytokines, which plays an important role in the initiation of inflammatory and immune response [15]. IL-6 is a proinflammatory cytokine secreted by macrophages, lymphocytes, mast cells, neurons, and glial cells. It also plays an important role in the development of RA [16]. In contrast, IL-10 is an important anti-inflammatory and immunosuppressive cytokine that is secreted by activated T cells, B cells, macrophages, dendritic cells, and mast cells [17, 18]. TNF-*α*, IL-1*β*, and IL-6 can cooperate to expand the conduction of inflammatory signals through positive feedback, and IL-10 usually acts as a negative feedback regulator in the inflammatory response. Large amounts of TNF-*α*, IL-1*β*, and IL-6 were found in the synovial fluid and serum of patients with RA. Therefore, inhibition of these proinflammatory cytokines has become a strategy for the treatment of RA, and then corresponding biological agents such as anti-TNF-*α* (adalimumab), anti-IL-1 (anakinra), and anti-IL-6 (tocilizumab) have been applied in the treatment of RA in clinical settings [19–21]. However, their use has increased the costs of treatment and the risk of serious adverse reactions such as infections and tumors [22]. Therefore, regulation of cytokine balance is more important than suppression inflammation [23]. The present research showed that EtOAc and CH_2_Cl_2_ could decrease the level of IL-1*β*, TNF-*α*, and IL-6 in the serum of AA rats, while they increase the level of IL-10, regulate the balance of cytokines, and improve the symptoms of AA.

In order to further clarify the pharmacodynamics material basis of these two effective parts, we decided to perform UHPLC analysis of the EtOAc and CH_2_Cl_2_ extracts and conducted a preliminary analysis of their components. The chromatogram showed that the retention time of the corresponding peak of the standard and sample was consistent. The results indicated that berberine and atractylodin were present in the samples. We speculate that they might be effective components in our experiments. However, there were other peaks in the samples, suggesting that the antiarthritic effect was the comprehensive effect of multiple components, which provided a sufficient experimental basis for further study of the active ingredients in EMS.

In conclusion, CH_2_Cl_2_ and EtOAc parts were verified to be the effective antiarthritic parts of EMS on the AA rat model. Downregulating the level inflammatory cytokines and upregulating anti-inflammatory cytokines may be the partial mechanism of the action of EMS. UHPLC analysis indicated berberine and atractylodin may be responsible for the antiarthritic activity of EMS. This research provided pharmacological and chemical foundation for the application of EMS in treating RA. However, the exact effect and mechanism of active ingredients of EMS need further investigation.

## Figures and Tables

**Table 1:** Effects of different polar solvent extracts of EMS on the change of body weight in AA rats.

Groups	Dose (mg/kg)	Day 0	Day 7	Day 14	Day 21	Day 28
Normal	—	180.27 ± 9.38	226.80 ± 11.17	255.52 ± 12.45	293.34 ± 11.80	321.07 ± 12.75
Model	—	180.95 ± 7.00	216.82 ± 8.22	237.15 ± 13.14^#^	235.25 ± 16.27^##^	249.9 ± 20.43^##^
PE	3 g/kg	179.74 ± 6.03	216.54 ± 13.67	239.9 ± 12.31	255.44 ± 18.46	258.76 ± 17.43
CH_2_Cl_2_	3 g/kg	180.56 ± 8.19	217.36 ± 11.22	242.8 ± 13.61	234.8 ± 13.31	257.35 ± 17.65
EtOAc	3 g/kg	180.31 ± 5.98	217.05 ± 14.00	239.1 ± 19.61	249.83 ± 20.92	273.53 ± 14.54
*n*-BuOH	3 g/kg	180.48 ± 4.62	218.51 ± 9.84	240.72 ± 16.27	238.84 ± 19.31	264.5 ± 19.91
MTX	0.5 mg/kg	180.42 ± 8.09	217.78 ± 10.45	238.57 ± 14.75	249.11 ± 20.11^##^	257.98 ± 18.31

^#^ *P* < 0.05 and ^##^*P* < 0.01 vs. the normal group (±SEM, *n* = 7).

## Data Availability

The data used to support the findings of this study are available from the corresponding author upon request.
